# Differences in Self-expression Reflect Formal Evaluation in a Fourth-year Emergency Medicine Clerkship

**DOI:** 10.5811/westjem.2016.11.31262

**Published:** 2016-12-19

**Authors:** Michael Chary, Amy Leuthauser, Kevin Hu, Braden Hexom

**Affiliations:** * New York Presbyterian/Queens, Department of Emergency Medicine, Flushing, New York; † University of Otago, Dunedin Hospital, Dunedin, New Zealand; ‡ Icahn School of Medicine at Mount Sinai, Department of Emergency Medicine, New York, New York; § Rush Medical College, Department of Emergency Medicine, Chicago, Illinois

## Abstract

**Introduction:**

Medical schools have begun to incorporate self-reflection exercises into their curricula, with the belief that these exercises help students master the material more deeply and perform better. Reflection may be a potential learning tool for emergency medicine (EM), but there are few data supporting this hypothesis. The authors evaluated the relationship between a linguistic marker of the degree of reflection after a student’s shift in an emergency department and that student’s clerkship performance.

**Methods:**

The authors conducted a retrospective case series by analyzing the performance and reflective statements of 116 students from a single medical school who participated in a required EM clerkship at one or two of four clinical sites from 2013–14. After each shift, an attending emergency physician evaluated the student according to the RIME (Reporter-Interpreter-Manager-Educator) scheme. The authors developed software to extract the text from those comments, remove uninformative words and standardize the remaining words. The authors determined the most common words and two-word phrases that students used to describe their shift. The correlation between students’ final clerkship grades and the fraction of student comments with at least one content word was analyzed.

**Results:**

Of the 145 possible students, 116 were included for analysis. The other 29 were excluded as they were visiting students who did not receive a final numeric grade. The correlation between final grade and the number of completed self-reflections was 0.32. The correlation between final grade and the average number of words in each self-reflection was 0.21. The first correlation is significantly greater than 0 (p=0.03, t-test), but the second correlation is not (p=0.16, t-test). The median final grade of those who wrote reflections on more than half of their shifts was significantly greater than those who wrote reflections half of the time, 83.675 versus 79.23 (p=0.05, 2-sample Kolmogorov-Smirnov test).

**Conclusion:**

Students who reflected more frequently received a higher grade in an EM clerkship for fourth-year medical students. The number of words in each reflection was not significantly correlated with grade performance. The most common words and phrases students wrote were associated with learning and managing patients.

## INTRODUCTION

Undergraduate medical education is becoming, increasingly, self-directed. Reflection is an essential aspect of self-directed learning.^1^ Reflection has been defined as “the process of analyzing, questioning, and reframing an experience to make assessment of it for the purpose of learning or to improve practice.”^2^ The hope is that forming personal and emotive connections with facts will reinforce those facts for the student. Students in clerkships often reflect upon their performance, but it is unclear if self-reflection leads to better academic performance.^3^ Structured reflection may be a useful teaching and learning tool in emergency medicine (EM).^4^ Reflection with standardized patients leads to better performance by students.^5^,^6^ Reflection may promote the development of professionalism and empathy in EM.^7^ We could find no study demonstrating that reflection leads to better patient care.^8^

The purpose of this study was to determine whether self-reflection in medical students correlates with academic performance in EM. We studied fourth-year medical students in a required EM clerkship. We used natural language processing to correlate the depth of reflection with clerkship performance in an objective and generalizable way.

## METHODS

We conducted a retrospective analysis of all students in a required EM clerkship at a single U.S. medical school from October 2014 to October 2015. Students completed 14 shifts while rotating in 1–2 of four hospitals - one city trauma center, two community hospitals, and one academic tertiary care hospital. All didactics were held together at a central location. The objectives and evaluation processes were uniform across sites. At the end of each shift, students were encouraged to complete a written reflective exercise in a logbook. The reflective exercise asked students the following:

“Reflect on your experience today. What was particularly challenging? Did you learn something unexpected? How will this change your practice for the future? Write a few sentences reflecting on your shift.”

An attending physician evaluated each student after each shift according to the Reporter-Interpreter-Manager-Educator (RIME) scheme, with “Educator” replaced with “Superior” in our evaluation tool. Students were required to submit these evaluations for grading by the end of the rotation. Additional components of the final grade included participation and final examination score. Students rotating from other institutions were not included.

### Software

All analysis was written by MC and performed in Python.^9^ Natural language processing was performed using the Natural Language Toolkit for Python, version 3.0.^10^ Bootstrapping and the calculation of Jaccard similarity were performed using NumPy/SciPy.^11^ Figures were made using the matplotlib plugin.^12^ Multinomial Naive Bayes classification was performed using NLTK and sci-kitlearn.^13^ All code used to analyze and generate the figures as well as supporting documents are available at the following repository: https://github.com/mac389/leuthauser.

Authors transcribed the booklets into a database. All medical abbreviations and contractions were replaced with their long form, and illegible comments were ignored. A de-identified version of the database is available at the repository referenced above. The text of each student comment was processed as follows:

1. Text converted to lower case
2. Comments tokenized into words
3. Stopwords removed
4. Remaining words lemmatized

### Stopwords

The term “stopwords” refers to words that occur frequently in a corpus but are unlikely to be informative. The removal of stopwords is a common preprocessing step in natural language processing to increase the sensitivity and specificity of analyses.^14^ The list of stopwords depends on the task. We used an amalgamation of the English stopwords list in the Natural Language Toolkit 3.0 package for Python and the 10,000 most frequently occurring words in the transcript of all episodes of *The Simpsons*. The list is available in our GitHub repository.

### Lemmatization

The term “lemmatization” refers to the mapping of all inflected forms of a word to a base form so that they can be analyzed as a single item. Words like “infect,” “infected,” “infection,” “infections,” become “infect.” To lemmatize words in our study we used the *WordNetLemmatize* function in NLTK 3.0. This function is a thin wrapper to WordNet’s *morphy* function, which removes all suffixes that occur in the WordNet database. *WordNetLemmatize* is more accurate if it is known which part of speech the word it is asked to lemmatize. For example, *patient* and *patients* should only be considered one item if *patient* is a noun. To identify the part of speech of each word, we used the *pos tag* function in NLTK 3.0. *Pos tag* is trained on the treebank corpus.^15^

#### Tokenization

The term “tokenization” refers to breaking a string of words into those words. Tokenization can be difficult when abbreviations and nonstandard punctuation are used. We used the *word tokenize* function in NLTK 3.0. This tokenizer uses regular expressions and is appropriate for pieces of text that do not have emoticons nor use contractions extensively.

#### Jaccard similarity

The “Jaccard similarity”^16^ quantifies the similarity between two sets of objects. The Jaccard similarity is defined as the ratio of number of objects two sets have in common to the total number of unique objects across both sets.

### Bootstrapping

In statistics, “bootstrapping” refers to a process of resampling without replacement to generate an empirical probability density function.^17^ It allows the estimation of the statistical significance of a parameter when the underlying distribution is not known. In this paper we use it to estimate the statistical significance of Jaccard similarities.

## RESULTS

### Demographics

**Figure 1:**
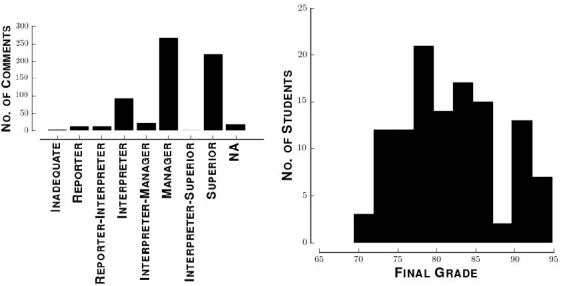
Demographics in a study looking at the impact of the frequency of medical students’ post-shift reflective comments on their final grade in an emergency medicine clerkship. Study demographics. Left: Distribution of attending ratings. Hyphenated ratings indicate that an attending circled two categories. Right: Distribution of final grades.

**Figure 2:**
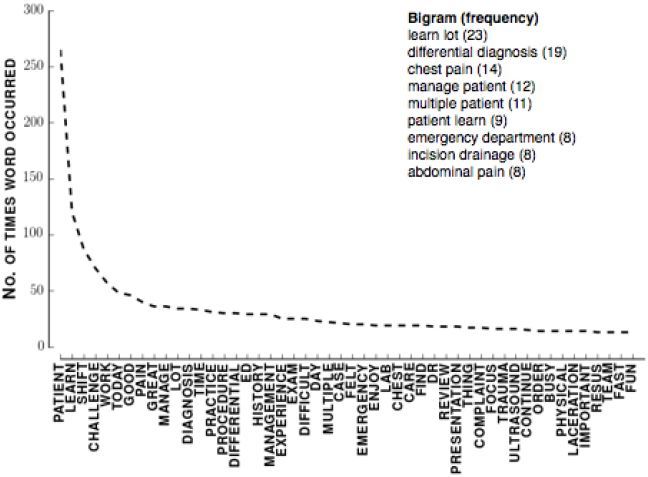
Left: Most common words in all student comments. Right: Most common bigrams.

Figure 1 shows the distributions of faculty ratings and final grades. Hyphenated categories refer to evaluations where the attending circled two adjacent categories evenly. Figure 2 shows the 50 most common single words (unigrams) and nine most common two-word phrases (bigrams) in all student comments that remained after processing. As an example, after processing the phrase *loss of consciousness* becomes (*loss*, *consciousness*). That phrase has two unigrams (*loss* and *consciousness*) and one bigram (*loss consciousness*).

### Correlation Between Reflection and Final Grade

**Figure 3:**
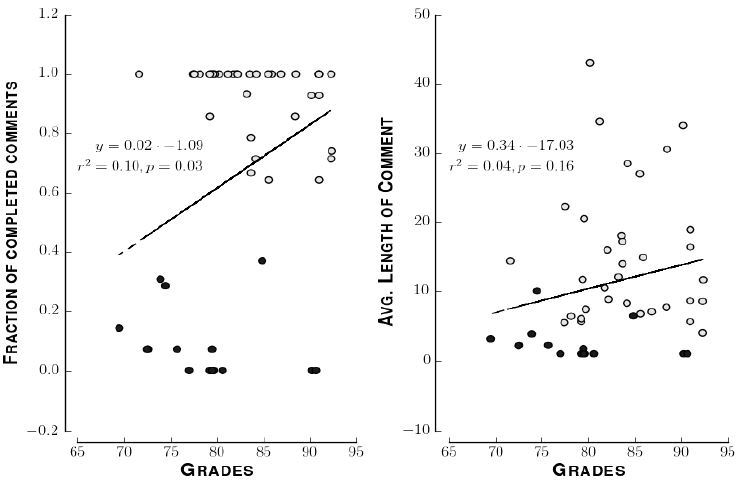
Correlation between clerkship grade and reflection. Left: Scatter plot of grades versus fraction of completed comments. Each point represents one student. Dashed line indicates regression of fraction of completed reflections against grades. Inset: Top line shows equation of regression line. Bottom line shows coefficient of determination and p-value that the slope of the regression line is significantly different from zero. Right: Scatter plot of grades versus average length of comments. Each point represents one student. Dashed line and inset indicate the same as in the left panel. In both panels solid circles represent those who completed less than half of the comments. Hollow circles represent those who completed more than half of the comments.

**Figure 4:**
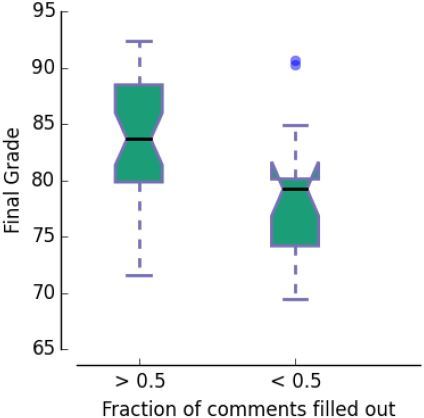
Comparison of final grade in students who commented on more than half of their shifts with those who commented on less than half. Tukey boxplot. Black horizontal line denotes median. Dimple denotes 95% confidence interval for median. Box denotes interquartile range. Whiskers denote 2nd and 97th percentiles. Dot indicates an outlier.

Figure 3 shows the correlation between the fraction of comments each student completed or the average lengths of each student’s comments and that student’s performance in the clerkship. We considered a comment completed if the comment had at least one legible word that was not a stopword. The correlation between the fraction of comments each student completed and clerkship grade was significant (two-tailed t-test; p=0.03). The correlation between the average length of comments and clerkship grade was not significant (two-tailed t-test; p=0.16). There are two clusters in the right panel of Figure 3. The cluster of hollow circles corresponds to students who commented on their experience more than half of the time. The cluster of solid circles corresponds to those who commented less than half of the time. The median final grades of the completers and non-completers, 83 +/−4 and 79 +/− 3 (median +/− interquartile range) are not significantly different, and the 95% confidence intervals for the medians overlap (Figure 4). The clusters do come from different distributions (Kolmogorov-Smirnov test; D statistic 0.5; p=.005) and the Jaccard similarity between the two was 0.06.

**Figure 5:**
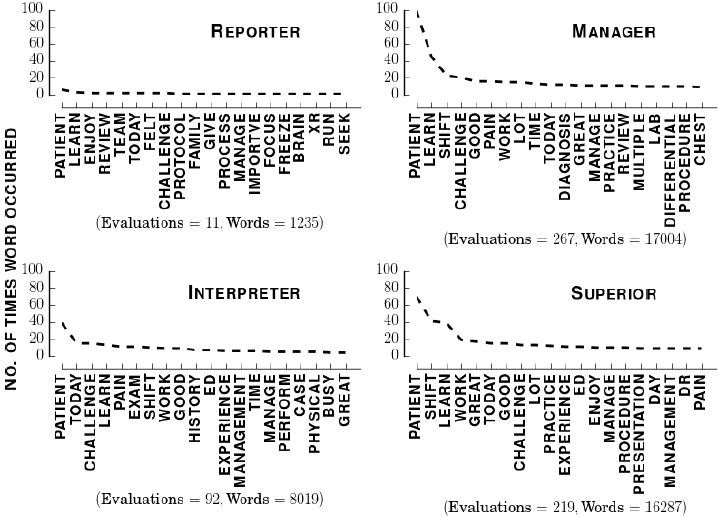
Most common words in each RIME category. Text in upper right of each inset denotes category. Label on x-axis details how many evaluations and total number of words used before lemmatization and removing stopwords.

Figure 5 shows the most common words in each RIME category. We excluded students rated as inadequate because they were too few. We also excluded hybrid categories. The Jaccard similarity between the pairs of panels in Figure 5 was the following: Reporter-Interpreter (0.989, p=0.436), Reporter-Manager (0.1494, p=0.2694), Reporter-Educator (0.1364, p=0.3166), Interpreter-Manager (0.3699, p<0.001), Interpreter-Educator (0.3158, p<0.01), Manager-Educator (0.4286, p<0.01). It suggests that those rated “Manager” used more words in common with those rated “Superior/Educator” than did those rated “Reporter” or “Interpreter.” Those rated “Reporter” used mostly different words to describe their experiences as compared with those rated “Interpreter,” “Manager,” or “Superior/Educator.”

## DISCUSSION

The main result of this study is that the number of comments medical students make after their shifts correlate with their end-of-rotation grades in an EM clerkship. Students who wrote more reflective or complex comments scored higher, although this increase was not statistically significant.

We propose several theories to explain these findings. As described previously, reflection has correlated with improved student performance with standardized patients.^5^ This may be extrapolated to the clinical setting although no previous studies have explored this hypothesis. Because students were evaluated on their clinical performance, and the reflection instructions prompted students to reflect immediately after their shift, they may simply have been trying to demonstrate diligence, even though their reflective comments were not included in the grading scheme. This suggests that some students who obtain higher clerkship grades do so because they are simply “completionists.” Final grades may still be more reflective of the completion of a list of tasks rather than the achievement of competencies, despite the move more generally in medical education towards a competency-based model.

Reflection has also been described to promote professionalism and empathy in EM.^6^–^7^ Professionalism and empathy are also explicit anchors used in our faculty evaluation and therefore are qualities upon which final grades are, in part, based. Furthermore, as the reflection exercise asked students to consider their most challenging cases of the day, this may have prompted students to review clinical material more than they otherwise might have done. Students who have been prompted to be more engaged with the material may therefore perform better in the clerkship. Finally, students who wrote more comprehensive and profound reflective pieces may tend to have attributes (i.e., stronger work ethic, greater attention to detail) that earn them better grades – these attributes could be confounders. Thus, the correlation does not necessarily imply causation.

A secondary result is that “Reporters,” “Interpreters,” “Managers,” and “Educators” use different words to describe their reflections.^18^ The word frequencies were tabulated once the attending evaluations were known. We could not determine the statistical significance of this association. The observations are not independent. Each student generated 14 comments and attending evaluations.

This study is the first to use natural language processing to quantify the relationships between how medical students perceive their performance, how faculty perceive their performance, and student performance on an objective measure, a final grade. Natural language processing has been used previously in more restrictive cases, such as to partially automate detecting when medical students have met procedural requirements.^19^ This suggests that undergraduate medical education can use natural language processing to quantify an important but as yet difficult pair of variables to operationalize, motivate, and reflect.

Quantifying the degree of reflection is challenging in medical school. What constitutes reflection changes as students progress through their education. Students may use similar words to describe different levels of reflection. Our study demonstrates a relationship between the pattern of exposition of medical students and their academic performance, these limitations notwithstanding.

## LIMITATIONS

The association between the reflection exercise completion and summative performance may simply reflect an underlying attribute, such as diligence. Counting comments with single legible words as “complete” may have diluted our statistical power. Students pursuing EM may perform better than students interested in other specialties. They may schedule the clerkship earlier to obtain letters of recommendation. With only one year’s data, we could not control for seasonality or specialty. Additionally, we did not control for the site in which a student rotated, which may have had effects on reflection, grading, or both.

It was unknown whether students wrote their reflections after each shift as instructed, or in bunches. Over 200 reflections were evaluated as “Superior.” Disproportionate representation of some categories at the expense of others makes it harder to find distinguishing features of each category. We excluded hybrid categories as it was unclear whether the evaluator intended to circle both categories, indicating performance between two categories. Excluding those comments may have decreased the power of the study. There was insufficient data to train a naive Bayes classifier to predict attending evaluations from student comments.

## CONCLUSION

Fourth-year medical students who reflected more frequently received a higher grade in a required EM clerkship. The number of words in each reflection was not significantly correlated with grade performance. A more formal reflection program could help identify students struggling to master the content before the end of the clerkship. More profound reflection may potentially augment student learning and clinical performance through increased self-engagement and awareness. However, future studies that target these particular outcomes need to be conducted.
